# Two-Step Progressive Transcrestal Sinus Augmentation Using a 4.5 mm Unloaded Implant as a “Temporary Implant” in Highly Atrophic Ridge: Case Report

**DOI:** 10.1055/s-0042-1755557

**Published:** 2023-01-25

**Authors:** Eduardo Anitua

**Affiliations:** 1 Private Practice, Eduardo Anitua Clinic, Vitoria, Spain; 2 University Institute for Regenerative Medicine and Oral Implantology, UPV/EHU - Eduardo Anitua Foundation, Vitoria, Spain; 3 BTI Biotechnology Institute, Vitoria, Spain

**Keywords:** sinus floor augmentation, short implants, bone atrophy

## Abstract

Severe atrophic posterior maxillary ridge (residual bone height < 3 mm) could be a challenging situation to place dental implants. Several treatment options have been proposed, but some of them may require advanced surgical skills to achieve best results. In this article, we present a novel and easier technique to allow implant placing in localized areas of severe atrophy. In a first step, a 4.5-length extra-short (unloaded) implant is placed after a transcrestal maxillary sinus floor augmentation (MSFA). After the gained apical bone consolidation, this “temporary implant” is atraumatically removed and a longer and wider definitive implant is placed to support the definitive single restoration. The case of a 45-year-old female treated with this approach is also presented. The patient suffered a severe resorption in the upper right molar area after a tooth extraction. Four months after the “temporary implant” placement and MSFA grafting with plasma rich in growth factors and autologous bone, 3 mm of dense apical bone gain could be observed. In a second surgical time, the 4.5 mm-length “temporary implant” was removed, and a 5.5 mm-length “definitive implant” was placed. This second implant was placed in a denser type 1 (1,000 Hounsfield Unit) new formed apical bone. Four months later, the implant was loaded with a screw-retained crown over a transepithelial (intermediate abutment). After 1-year follow-up, the implant was in health and no mechanical or biological complications were noticed. The satisfactory results of this case encourage the realization of new studies to elucidate its reproducibility.

## Introduction

Bone and soft tissue deficiencies at implant sites may result from a multitude of factors. 1 After tooth extraction, the alveolar bone undergoes natural resorption processes both vertically and horizontally. 2 In the posterior maxilla, the presence of the maxillary sinus along with a scarce residual bone may hinder implant housing resulting in the need for auxiliary techniques to place implants. Several methods have been applied to address this problem, such as short implants, zygomatic implants, regular size tilted implants, maxillary sinus floor augmentation (MSFA), and bone grafting. 3 4 5

Different MSFA techniques have been broadly used with high success rates. 6 7 The sinus lift can be performed through a lateral window or a transcrestal approach, placing the implants in the same surgical time or in a second stage. Lateral approach was introduced by Boyne and James in 1980 8 and it is still a well-documented and widely used technique. To get a less invasive and time-consuming method, Tatum 9 described in 1986 the first transcrestal approach, lifting the sinus membrane by fracturing the sinus floor. Summers in 1994 modified this technique by introducing the use of a kit of specific osteotomes. 10 Anitua et al 11 have performed transalveolar sinus lift using drills instead of osteotomes. Since then, high survival and success rates have been reported for other modifications of the original techniques subsequently developed. 4 6 12 13 The use of different grafting materials could influence the outcomes of MSFA as different systematic reviews have reported. 14 15 Regardless the followed surgical approach, MSFA is broadly considered to date a reliable procedure in the partially and fully edentulous maxilla. 6

The development of short implants has made it possible to avoid the need for MSFA in cases over 8 mm of residual bone 16 and to simplify the procedure in cases under 8 mm Furthermore, combination of short implants and transcrestal MSFA can be effective in the treatment of posterior maxilla with a mean residual bone height less than 5 mm. 11 17 The lateral approach was formerly suggested when the bone height is less than 5 mm, 18 but recently comparable outcomes have been observed in cases of less than or equal to 3 mm of residual bone height, regardless the followed implant placement technique (lateral or transcrestal, 1-stage or 2-tage). 19

The need for placing a dental implant in areas of posterior maxilla with severe vertical resorption (<3mm) can be a challenging clinical situation, particularly when the anatomy is not favorable, and the bone density is very low. The insertion of dental implants has shown to improve the bone density and thus its quality. This could not be achieved by only MSFA.

In this work, we present a novel way to face this clinical situation based on the use of an unloaded standard 4.5 mm-length extra-short implant as a “temporary implant” to perform a two-step transcrestal progressive MSFA. The objective of this technique was to achieve vertical bone augmentation with simultaneous increase in the residual bone density and quality. After the osseointegration of the 4.5 mm-length implant and the consolidation of the new formed apical bone, the implant was removed in a second surgical time to place a longer and wider implant. The “definitive implant” could be apically anchored in a denser new formed bone that contributed to achieve primary stability. To the best of our knowledge, this is a novel indication for extra-short implants.

## Case Presentation

A 45-year-old female patient was referred to our clinic due to the presence of severe pain at the upper right first molar area. The patient was in good general health.

During the exploration, the presence of enamel micro-cracks, masseter hypertonicity, and incisal attrition suggested bruxism. The upper right first molar showed deep vestibular probing, so a cone-beam computed tomography (CBCT) was performed to assess a suspected vertical fracture. Besides confirming the fracture, the radiological study showed the presence of a previous endodontic treatment and an apical radiolucency at the distobuccal root. This radiolucency had reabsorbed the thin cortical resulting in a buco-sinusal communication. A deep root protrusion into the maxillary sinus (type 3) 20 was also observed. The tooth extraction was carefully performed trying to preserve the scarce remaining bone. After the curettage of the inflammatory apical lesion, plasma-rich in growth factors (PRGF-Endoret, BTI Biotechnology Institute, Vitoria, Spain) was used to favor socket preservation. 21 22 23 24 The preparation protocol is explained in the following text.

**Fig. 1:**
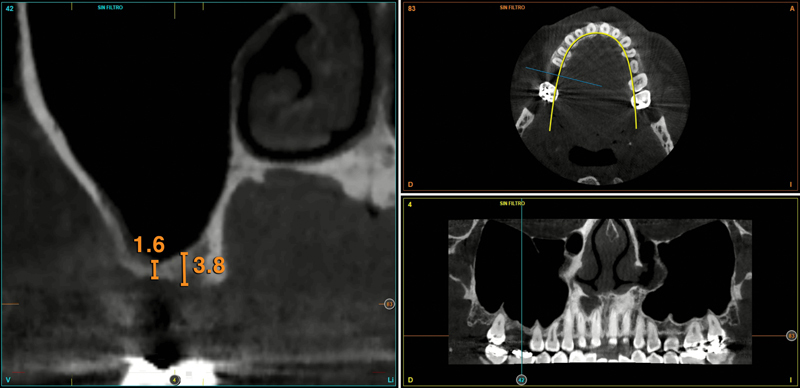
Cone-beam computed tomography image. Residual ridge measurements before the 4.5-length “temporary” implant placement. Note the discrepancy between palatal and buccal areas.

After explaining the details of the planification, the patient gave her informed consent. Four months after the dental extraction and bone regeneration, a new CBCT scan was obtained to assess the residual bone volume and quality, and to facilitate the implant surgery planning. Ridge height at the extraction zone was 1,6 mm at the buccal side and 3.8 mm in the palatal area ( Fig. 1 ). Bone density was under 200 Hounsfield Unit (HU; type IV bone). 25 A transcrestal MSFA was then planned to allow the placement of an extra-short 4.5-length implant (diameter 5.5). Given the discrepancy in residual bone height between the palatal and buccal areas, the implant positioning tried to get partial apical anchorage in the palatine zone.

**Fig. 2:**
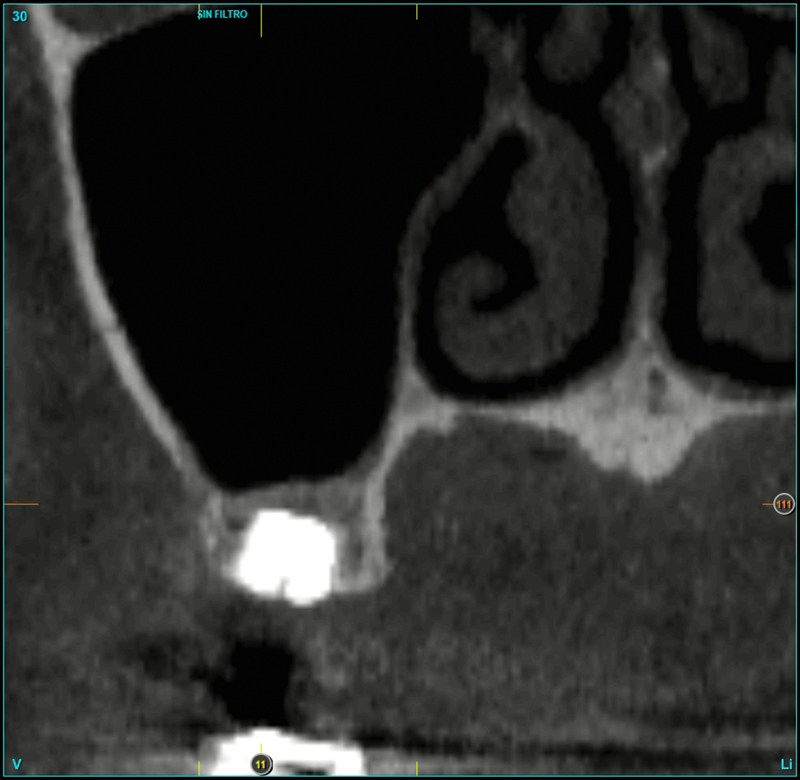
Cone-beam computed tomography image. Five months after the 4.5-length “temporary” implant placing. Three mm of bone gain over the implant apex could be observed.

**Fig. 3:**
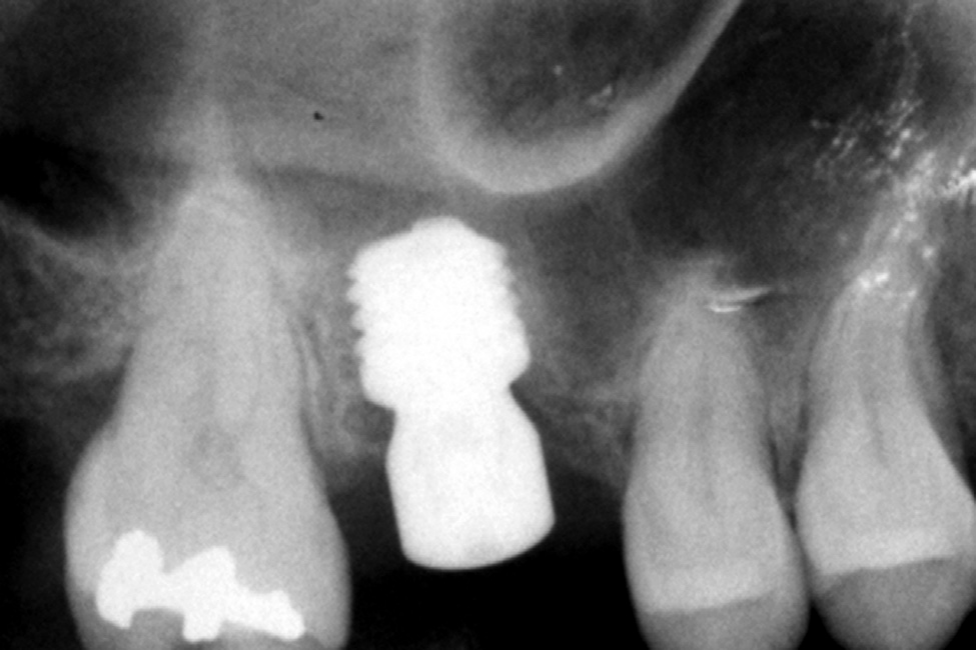
Panoramic image after the 5.5-length “definitive” implant placing.

**Fig. 4:**
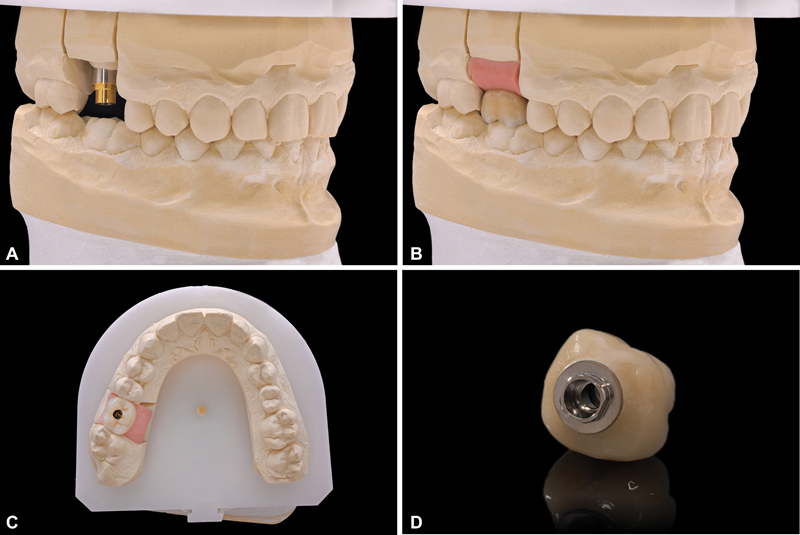
Composition: Prosthodontic rehabilitation detailed. The screw-retained crown was performed over a transepithelial (intermediate abutment). The suprastructure was designed and manufactured by CAD-CAM, covered with ceramic, and cemented to a Ti-interface.

Four months later, 3 mm of dense (1,000 HU) bone gain over the implant apex could be observed ( Fig. 2 ). At this point, the 4.5-length “temporary” implant was removed employing the counter-torque technique. The Kexim explantation Kit (BTI Biotechnology Institute, Vitoria, Spain) was used to atraumatically perform the explantation. In the same surgical time, a new 5.5-length implant was placed (diameter 6.0) to support a screw-retained single crown over a transepithelial (intermediate abutment). The definitive implant was loaded after 4 months of the “definitive implant” placing ( Figs. 3 and 4 ).

**Fig. 5:**
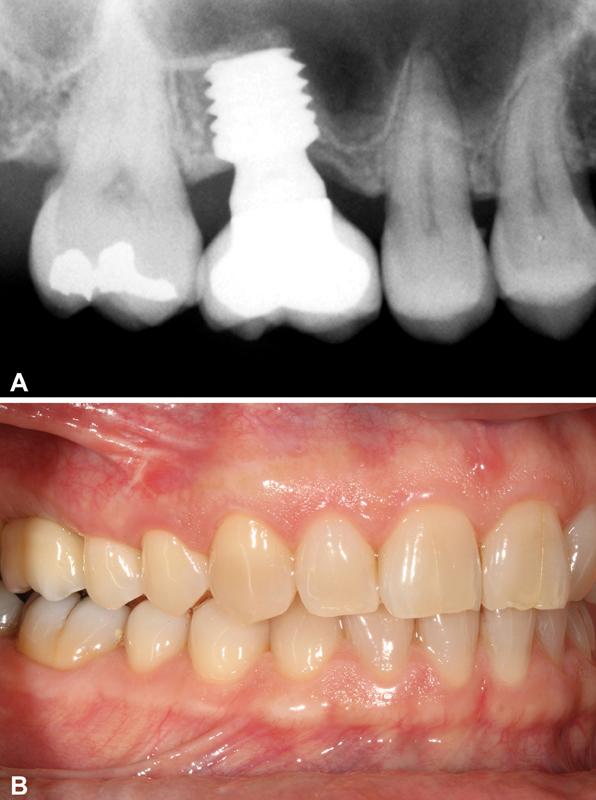
Composition: Panoramic image and clinical image 1 year after implant loading.

**Fig. 6:**
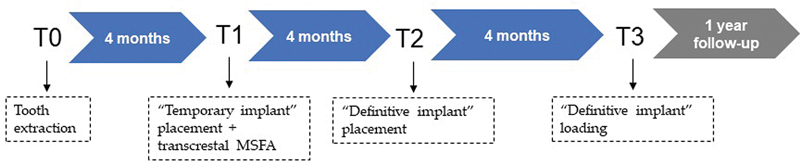
Timeline of the treatment. MSFA, maxillary sinus floor augmentation.

After 1-year follow-up, the implant was in health. No mechanical or biological complication was noticed, and the patient was totally satisfied with the results of the treatment ( Figs. 5 and 6 ).

### Drilling Protocol and Sinus Augmentation

Under local anesthesia, a crestal incision was practiced and a full-thickness flap was reflected to expose the alveolar crest. Both “transitional” and “definitive” implants were inserted following the low-speed drilling technique described by Anitua et al. 26 This drilling procedure was designed to preserve the peri-implant tissue and to allow collecting autologous bone. Subsequent diameter drills were employed without irrigation and low speed (up to 125 rpm). Lastly, the socket was wetted in PRGF (Fraction 2, Endoret-PRGF, BTI Biotechnology, Vitoria, Spain) and the implant was placed at a 15 to 20 rpm speed without irrigation.

**Fig. 7:**
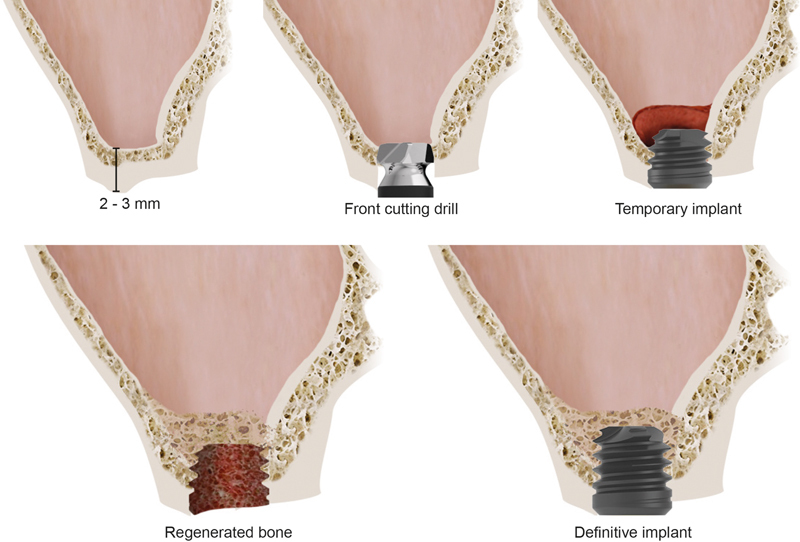
Maxillary sinus floor augmentation (MSFA) and 4.5 extra-short implant placing technique protocol.

To place the 4.5 extra-short “temporary implant” and to perform the MSFA, the transcrestal approach proposed by Anitua et al 17 was employed. The bone drilling was performed in two phases; in the first phase, conventional twisted bone drills were used. The working length (drill penetration into bone) was set at 1 mm shorter than the residual bone height. Then, in a second phase, the last 1 mm of the residual bone was gently prepared with a frontal-cutting drill ( Fig. 7 ). Once a small window was opened in the sinus floor, a fibrin membrane (Fraction 1, Endoret-PRGF, BTI Biotechnology, Vitoria, Spain) was introduced to protect the Schneiderian membrane. With the help of a blunt instrument, the membrane was raised and the space beneath the membrane was filled with a mixture of PRGF (Fraction 2) and autologous bone.

### Protocol for Obtaining PRGF Autologous Graft

Autologous PRGF was employed obtain the fibrin membrane and the sinus augmentation graft. For the preparation of both grafts, an Endoret-PRGF kit was used (KMU15, BTI Biotechnology Institute, Vitoria, Spain). Eighteen milliliters of the patient's own blood were processed according to the manufacturer's instructions. 27 The volume of plasma obtained was fractionated into Fraction 2 (F2) defined as the first 2 mL of plasma just above the buffy coat and Fraction 1 (F1) defined as the plasma volume above the F2. This allowed to prepare a total volume of 4 mL of F2 plasma. Platelet activation was performed by adding 10% calcium chloride.

## Discussion

We present the results of treating a severe localized bone atrophy in the posterior maxilla by a novel procedure. Several techniques and grafting materials have been proposed to allow implant placing in this scenario. Residual bone height, width, and quality, sinus anatomy, or the buccal-palatal bone wall distance could influence the selection of the most suitable treatment option. Nevertheless, this choice remains mostly based on the experience and skills of the clinician. 28

This novel two-step technique presented successfully achieved vertical bone augmentation and residual bone density enhancement. It allowed implant placing in highly resorbed posterior maxillary area with a simple and minimally invasive procedure. In the first step (first surgical time), a transalveolar MSFA was performed aiming to gain 1.5 to 3 mm of new formed bone and a 4.5-length extra-short implant was placed. No further biomaterial than autologous PRP and autologous bone was employed. After the osseointegration of the 4.5 mm-length implant and the consolidation of the new formed apical bone, the implant was removed in a second surgical time to place a longer and wider implant. The “definitive implant” could be apically anchored in a denser new formed bone that could contribute to achieve primary stability. The dimensions of this second implant were more appropriate to support the definitive restoration in a predictable manner. Nevertheless, the placement of an implant with the dimensions of the “definitive” implant since the beginning of the treatment, would have required more complex procedures.

This approach offers diverse advantages in scenario like the above presented. A lateral window opening is avoided, resulting in minimal trauma and reduced invasiveness and lower postoperative morbidity. 29 The latter is further facilitated by not using nonautologous biomaterials. The use of extra-short implants prevents the need for large volume grafts to create enough amount of new formed bone to house standard-length implants. This reduction in the volume to be grafted can also prevent complications (such as membrane perforation) and minimize the modification of the original sinus volume. In cases of remarkable discrepancy between the height of the residual bone in the buccal and lingual aspect, the insertion of a 4.5 extra-short and a minimal MSFA at the same time is particularly less complex and surgically timesaving than other treatment options. In the above presented scenario, this procedure is likely to be less demanding in terms of advanced surgical skills for the clinician than the procedure required to place a longer implant since the first surgical time. The use of front-action burs instead of osteotomes could be less discomforting for the patient during the surgery and provide safety and predictable results. 17

In relation to the grafting procedure, it has been stated that the volumetric stability of autogenous bone grafts in MSFA could be improved with addition of xenograft compared with autogenous bone. 15 Nevertheless, grafting with autogenous bone alone in MSFA could improve histomorphometry outcomes compared with other grafting materials. 30 Combination of anorganic bovine bone (ABB) and PRGF can improve the osteoconductive properties of ABB by increasing the volume of new bone formed. 31 PRGF alone or in combination with autologous bone has algo shown satisfactory results and predictability in MSFA. 16 32 Since this technique does not require large volume of bone gain, it can be performed using PRGF alone or in combination with particulate autologous bone when slightly higher growth is required.

The limitations of this procedure are the need for two surgical times before the implant loading and the total length of the treatment. Treatment costs could be also arguable since two implants are needed. However, the last can be offset by savings in bone substitutes, membranes, and other biomaterials.

Temporary, transitional, provisional, or interim implants and mini-implants were defined as a type of dental device that can be used during a defined time (normally the healing period) to maintain the bone volume or even to support interim removable prostheses. 33 Although they seem to be synonyms, the differences in the use of these terms can be rather confusing. Mini-implants 34 (narrow one-piece temporary implants) have been broadly used as solution to restore patient's masticatory function during the healing period of the “definitive implants.” “Expander implants” are other sample of transitional/temporary/provisional implants employed to maintain the achieved bone expansion in two-stage ridge-splitting expansion techniques. As in the present case report, the use of unloaded extra-short (regular) implants as a provisional solution to help in the graft stabilization has rarely been reported.

## Conclusions

Posterior maxillary localized severe bone atrophy (< 3mm residual bone), particularly in cases of low bone quality, could be successfully treated using this two-step progressive MSFA using an unload extra-short transitional implant. The satisfactory results achieved in this patient encourages to further explore the indications and long-term results of this novel technique.
